# A Unique Mutation in the Cystic Fibrosis Transmembrane Conductance Regulator (CFTR) Gene Causing Cystic Fibrosis in a Pakistani Child: A Case Highlighting the Need for More Awareness

**DOI:** 10.7759/cureus.54627

**Published:** 2024-02-21

**Authors:** Sinan Yavuz, Basil Elnazir, Saista Amin, Amal Sherif, Safiya Saif, Nader Francis

**Affiliations:** 1 Pediatrics/Pediatric Pulmonologist, Al Qassimi Women's and Children's Hospital, Sharjah, ARE; 2 Pediatrics, Children's Health Ireland (CHI) at Tallaght University Hospital, Dublin, IRL; 3 Pediatrics, Trinity College Dublin, Dublin, IRL; 4 Pediatric Gastroenterology, Al Qassimi Women's and Children's Hospital, Sharjah, ARE; 5 Pediatrics/Pediatric Consultant, Al Qassimi Women's and Children's Hospital, Sharjah, ARE; 6 Pediatrics/Pediatric Pulmonology, Al Qassimi Woman's and Children's Hospital, Sharjah, ARE; 7 Pediatrics/Pediatric Pulmonology, Al Qassimi Women's and Children's Hospital, Sharjah, ARE

**Keywords:** muconium ileus, pediatrics, pulmonology, cftr gene mutation, cystic fibrosis (cf)

## Abstract

Cystic fibrosis (CF) is a recessively inherited disease most commonly seen in Caucasians. The mutations in the cystic fibrosis transmembrane conductance regulator (CFTR) gene are responsible for the condition, and to date, more than 2000 mutations have been published in the literature. The most common mutation worldwide is F508del.

Here, we reported a five-year-old child who presented to the clinic with a chronic cough. Her newborn screening for CF was negative, including 139 mutation panels done in India. The sweat chloride test was positive, and CF gene sequencing was reported as c.2489dup p. (Glu831GLYFS *5) homozygotes mutation in the CFTR gene (Online Mendelian Inheritance in Man (OMIM) *602421). To the best of our knowledge, this gene was first described and published in the literature.

## Introduction

Cystic fibrosis (CF) is a rare autosomal recessive inherited genetic disorder that is common in Caucasians, affecting 1/2500 live births, with the carrier rate of the disease ranging from 1/26 to 1/30 [1]. The exact prevalence in non-Caucasian people is unknown, but the patient is increasingly recognized and diagnosed in this group [1-3]. The most commonly affected mutation worldwide is F508del, and the majority of patients who have been diagnosed at an early age will have at least one of these mutation copies. In adult life, most patients diagnosed with CF will have a milder disease [4,5]. The mechanism in cystic fibrosis is a disturbance in the transport of electrolytes across the epithelial cell due to dysfunction of the CF transmembrane conductance regulator (CFTR) [6]. This ultimately manifests as obstructive lung disease, pancreatic insufficiency, and elevated sweat chloride (epithelia of the respiratory tract, exocrine pancreas, intestine, male genital tract, hepatobiliary system, and exocrine sweat glands) [6,7].

We described a child diagnosed with CF at the age of six years with a rare genetic homozygote mutation, which revealed a c.2489dup p. (Glu831GLYFS *5) mutation in the CFTR gene (Online Mendelian Inheritance in Man (OMIM) *602421). To the best of our knowledge, this mutation is the first reported as a disease-causing mutation in CF.

## Case presentation

A five-year-old girl with a known case of suspected CF was referred to our clinic due to a persistent wet cough. She was born at term by elective caesarian section with a birth weight of 2.97 kg. An early post-natal abdominal distension delayed meconium passage was noticed at birth, and an X-ray of the abdomen, and gastrografin study showed microcolon. The patient was diagnosed with meconium ileus and required surgical intervention on two occasions. The first was laparotomy with partial ileal resection and ileostomy (resection of 20 cm of the terminal ileum, appendicectomy with ileostomy). Then, the second surgery was done after one month, which was the closure of the ileostomy and resection of part of the ileum and colon (the length of resected parts not determined). After that, the ileostomy opened by itself again; the patient was kept in another Hospital for more than two months. During this duration, she received many antibiotics, antifungals, and acetyl cystine injections in the ileostomy opening. The patient was referred to our hospital at that time for further management.

The patient started to have cholestasis with low platelets and high creatinine observed with anuria. During the admission and in view of the history of meconium ileus and high IRT, a sweat test was done and reported as positive with a sweat chloride test of 114 mmol/L. Suspicion of CF was raised, and a genetic study for 139 cystic fibrosis transmembrane conductance regulator (CFTR) genes was sent, which was surprisingly reported as normal. 

The child was followed up by the gastroenterology team as her initial presentation was gastrointestinal (GI) rather than a CF-related disease, and she was started on a feeding plan (gradually introduced) with partial total parental nutrition (TPN). The ileostomy stoma was subsequently closed. After reaching the full feed. The child was discharged with a follow-up in the gastroenterology clinic. During the follow-up, she had thrombocytopenia and hepatosplenomegaly with signs of portal hypertension, so an Upper GI endoscopy showed the development of esophageal varices grade 1. During the follow-up, she continuously had a cough, and then the patient was referred to the pulmonology clinic. 

**Figure 1:**
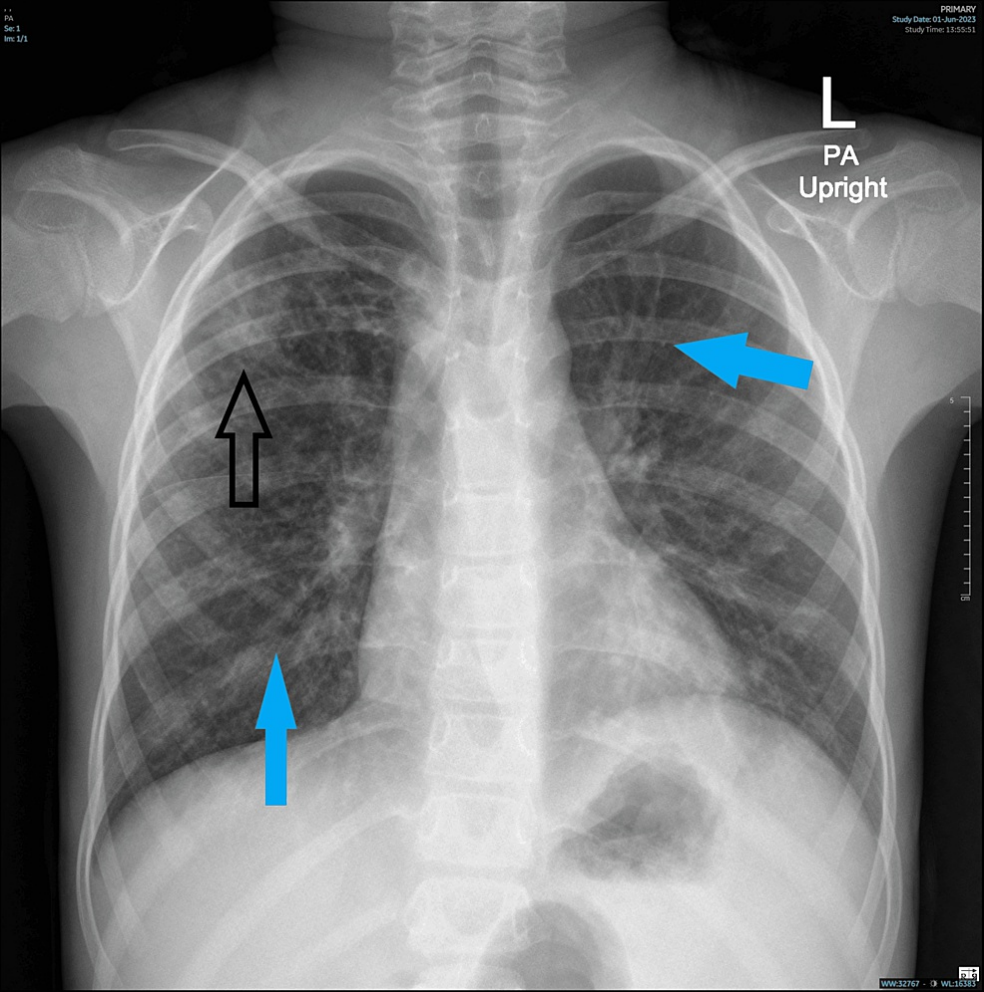
Chest X-ray image The chest X-ray showed patchy air space opacity in the right upper lung zone, likely infective in nature (black arrow). Bilateral accentuated broncho-vascular marking with minimal peri-bronchial thickening (blue arrow).

When she presented to our clinic, her clinical examination revealed finger clubbing, chest bilateral fine crepitation, and abdomen hepatosplenomegaly. A chest X-ray was ordered and showed right upper lung zone patchy air space opacity likely infective in nature. Bilateral accentuated broncho-vascular marking with minimal peri-bronchial thickening (Figure 1).

**Figure 2:**
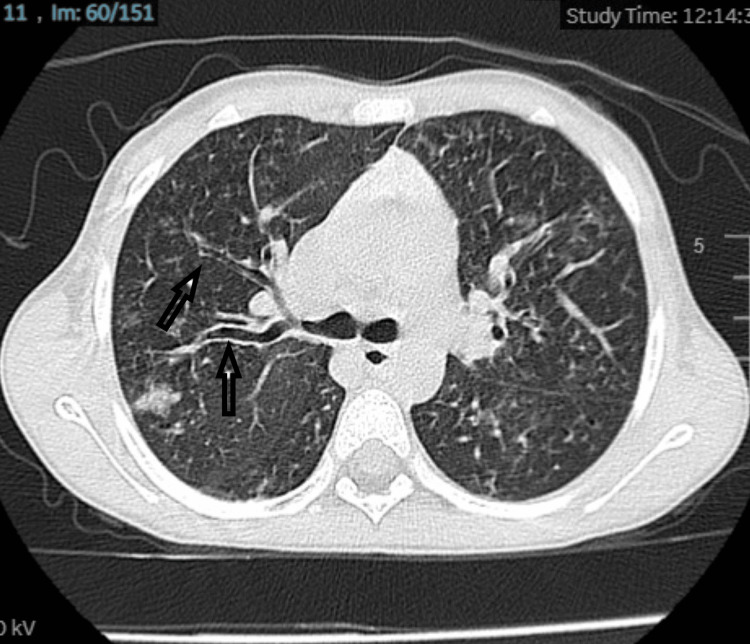
CT image of the chest CT chest revealed a tree in the bud with tubular bronchiectasis (black arrow).

Her blood investigation total blood count (CBC), C-reactive protein (CRP), electrolytes, and minerals were normal. Sweat test repeated and came high 114 mmol/L, Liver function test was slightly elevated. Stool elastase was <200 mcg/g. Computed tomography (CT) chest showed a tree in the bud with tubular bronchiectasis (Figure 2).

Bronchoscopy showed bilateral profuse mucopurulent secretions. Bronchoalveolar lavage subsequently grew pan-sensitive *Pseudomonas aeruginosa*. Full CFTR gene sequencing identified a novel mutation, CF apparent c.2489dup p. (Glu831GLYFS *5) mutation in the CFTR gene (OMIM *602421). Method genomic data of the patient was fragmented, and the coding exons of the CFTR gene, as well as the corresponding exon-intron boundaries, were enriched using Roche/KAPA sequence capture technology and sequenced simultaneously by Illumina technology using an Illumina system. The requested gene panel was extracted from whole gene sequencing (WES) data. The target regions were sequenced with an average coverage of 110-fold. For 100 % of the regions of interest, a 15-fold coverage was obtained.

Currently, the child is on sodium chloride 5% nebulizer twice daily, salbutamol nebulizer, vitamins (A, D, E, and K), Azithromycin three days per week, and chest physiotherapy. She is in stable condition with regular follow-ups in the clinic.

## Discussion

Although knowledge about fibrocystic disease has increased in recent decades, the genetic and clinical manifestations remain various. In most centers, the diagnosis of CF via newborn screening is followed by confirmatory tests, which are sweat and genetic tests [8]. CFTR gene is responsible for more than 85% of mutations, and more than 2000 mutations have been identified [9].

The genetic mechanism has become clear in the development of meconium ileus (MI), and it occurs in 13- 17% of patients with CF disease [10,11]. The most common CFTR gene associated with MI is homozygotes F508 [11]. Recent studies showed no differences in survival between patients with MI and with non-MI [12,13]. There are many studies done to know the relationship between MI and CF-related liver disease. Some of these studies show an increased risk, some with no difference in risk, and some with a decreased risk of MI with patient CF-related liver disease [12,14-18].

Our case had a c.2489dup p. (Glu831GLYFS *5) mutation in the CFTR gene (OMIM *602421), and it is the first mutation identified in the CF database. This gene leads to frameshift, resulting in a premature stop codon and subsequent mRNA degradation (nonsense-mediated decay), or truncation of protein. Interestingly, the first genetic study for the 139 gene was negative, but repeated Whole Exome Sequencing (WES) came positive for CF. She presented with many CF manifestations, but to date, we are not sure if it is related to CF because it started post-delivery, especially liver disease. Also, at that age, she developed meconium ileus, pancreatic insufficiency, and abnormal sweat test. She is six years old now and doing well, and it is too early to predict the prognosis of her disease. This case is not the first rare CF mutation reported in this region [3,19].

Delayed or missed diagnoses of patients have a bad effect on the outcome. Studies showed that initiation of early management and treatment is necessary to improve the course of the disease, with the best outcome if it is done as early as the age of twp months [20]. In this case report, we want to emphasize that when there is a high suspicion of CF, doctors must continue investigations to rule out cystic fibrosis.

## Conclusions

CF is a common disease in Caucasians and has an estimated occurrence of 1/2500 live births. The exact prevalence of CF is not known in Asia and the Middle East because of a lack of awareness and investigation. This generally leads to unnecessary and deleterious delays in diagnosis. Children in this area are not diagnosed, or even if diagnosed, the gene type is not published, causing a lack of knowledge about the most common mutations and other rare mutations in this area. The outcome of CF is better as early as the disease is diagnosed. We presented here a c.2489dup p. (Glu831GLYFS *5) homozygotes mutation in the CFTR gene (OMIM. *602421), which is the first time published in the literature.
